# Chloride Voltage-Gated Channel 2 (CLCN2)-Related Leukoencephalopathy Exhibiting Reduced Choline Levels on Magnetic Resonance Spectroscopy

**DOI:** 10.7759/cureus.61716

**Published:** 2024-06-05

**Authors:** Kenta Ochiai, Takashi Ohashi, Harushi Mori, Hirotomo Saitsu, Jun-ichi Takanashi

**Affiliations:** 1 Department of Pediatrics, Tokyo Women’s Medical University Yachiyo Medical Center, Yachiyo, JPN; 2 Department of Neurology, Kamagaya General Hospital, Kamagaya, JPN; 3 Department of Radiology, Jichi Medical University, School of Medicine, Tochigi, JPN; 4 Department of Medical Science, Hamamatsu University School of Medicine, Hamamatsu, JPN; 5 Department of Pediatrics, Tokyo Women's Medical University Yachiyo Medical Center, Yachiyo, JPN

**Keywords:** magnetic resonance spectroscopy, magnetic resonance imaging, choline, leukoencephalopathy, clcn2

## Abstract

In this article, we report the third case of chloride voltage-gated channel 2 (CLCN2)-related leukoencephalopathy (CC2L) in Japan. The patient presented with headache, vertigo, and mild visual impairment. The *CLCN2* variant of the patient, NM_004366.6:c.61dup, p.(Leu21Profs*27), was also found in two other Japanese patients as this variant is relatively common in the Japanese population. Magnetic resonance imaging (MRI) revealed T2 prolongation with reduced diffusion in the bilateral posterior limbs of the internal capsule, cerebral peduncles, and superior and middle cerebellar peduncles. Magnetic resonance spectroscopy (MRS) of normal-appearing white matter revealed decreased choline content. This represents the first evidence of decreased choline levels in CC2L, highlighting the superior sensitivity of MRS over MRI.

## Introduction

Chloride voltage-gated channel 2 (*CLCN2*)-related leukoencephalopathy (CC2L) is a rare autosomal recessive disorder caused by pathogenic variants in *CLCN2*. CC2L is characterized by nonspecific neurologic findings, mild visual impairment from chorioretinopathy or optic atrophy, male infertility, and characteristic findings on brain magnetic resonance imaging (MRI) [1]. In this study, we described a Japanese patient with CC2L in whom magnetic resonance spectroscopy (MRS) provided the first evidence of reduced choline (Cho) levels, which might reflect the pathogenic condition of intramyelinic edema.

## Case presentation

**Figure 1:**
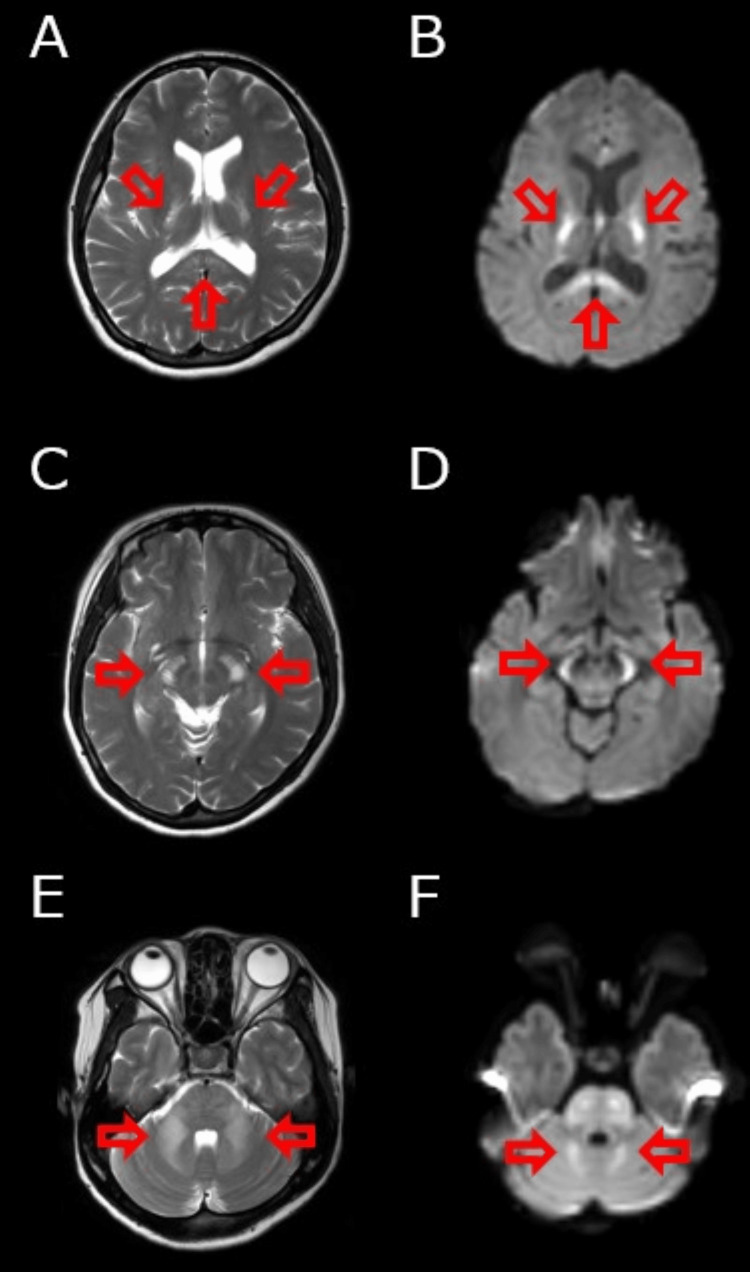
Brain magnetic resonance imaging of the patient at the time of presentation Hyperintensities on axial T2-weighted imaging (A, C, E) and diffusion-weighted imaging (B, D, F). Abnormal signals in the bilateral posterior limbs of the internal capsule and splenium of the corpus callosum (A, B), cerebral peduncle (C, D), and superior cerebellar peduncles (E, F).

The case involved a 32-year-old woman who was born to healthy and nonconsanguineous parents and had no prior medical history. Her family history was unremarkable. She had headaches, vertigo, and visual impairment at night since her 20s. She visited our hospital because her headache and vertigo worsened. Her tandem gait was poor, but she had no other abnormal neurological findings. Brain MRI revealed T2 prolongation with reduced diffusion in the bilateral posterior limbs of the internal capsule (Figures 1A, 1B), cerebral peduncles (Figures 1C, 1D), and superior and middle cerebellar peduncles (Figures 1E, 1F).

**Figure 2:**
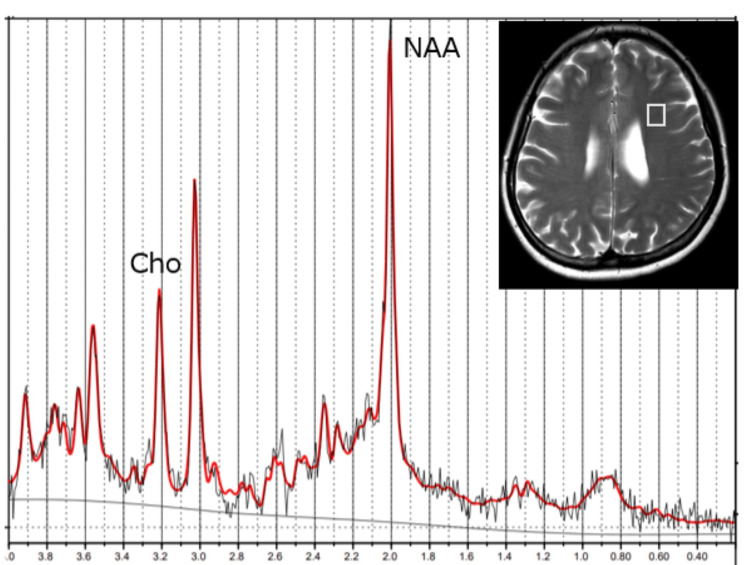
Magnetic resonance spectroscopy of the patient at the time of presentation Magnetic resonance spectroscopy of the left parietal white matter revealed decreased choline and N-acetyl aspartate levels. Cho: choline, NAA: N-acetyl aspartate

Single-voxel MRS of normal-appearing frontoparietal white matter (voxel size of 4.5 cm^3^) revealed reduced Cho (1.14 mM; age-matched control, 1.57 ± 0.11 mM (mean ± SD)) and N-acetyl aspartate (NAA) levels (7.60 mM; age-matched control, 9.3 ± 0.2 mM) (Figure 2).

MRS was performed with point-resolved spectra using repetition time (TR) of 5000 ms, echo time (TE) of 30 ms, and number of excitations (NEX) of 32. Quantification of metabolites, including NAA and Cho, was performed using Linear Combination of Model (LCModel) method. Bilateral prolonged latency of the P100 wave (124-127 ms) in the visual evoked potential was detected. Her headaches and vertigo persisted; however, she could perform her daily activities without major limitations.

**Figure 3:**
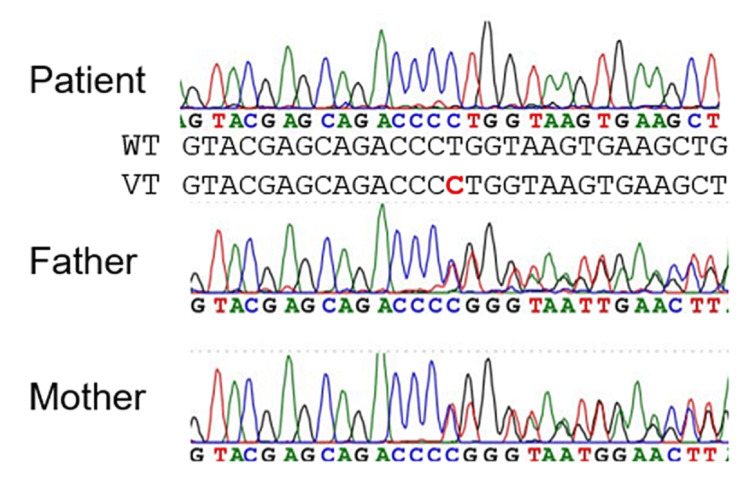
CLCN2 sequencing of the patient’s family *CLCN2* sequencing of the patient’s family. Frameshift mutation (c.61dup, p.Leu21Profs*27) is shown in *CLCN2*. The patient has a homozygous mutation. CLCN2: Chloride voltage-gated channel 2.

This study was approved by the Institutional Review Board Committees of Tokyo Women’s Medical University (approval number 356, 3535R) and Hamamatsu University School of Medicine (approval number 20-207). After obtaining written informed consent, genomic DNA was extracted from the blood leukocytes of the patient and her parents. For whole-exome sequencing (WES), the patient’s DNA was captured using a Twist Exome 2.0 panel (Twist Bioscience, South San Francisco, CA, USA) and sequenced on a NovaSeq6000 (Illumina, San Diego, CA USA) with 100-bp paired-end reads. Alignment of the sequenced reads to the reference genome (GRCh38), and variant calling were performed using the fq2bam and DeepVariant software packages of Clara Parabricks v4.2.0 (NVIDIA Corporation, Santa Clara, CA, USA). We focused on rare variants, which were defined by a minor allele frequency of ≤0.01 in an in-house control exome database (123 control individuals), the Japanese Multi-Omics Reference Panel (https://jmorp.megabank.tohoku.ac.jp/), and the gnomAD v4.0.0 database (https://gnomad.broadinstitute.org/). WES revealed a homozygous frameshift variant in *CLCN2* NM_004366.6:c.61dup, p.(Leu21Profs*27), leading to a diagnosis of CC2L. Both father and mother were heterozygous carriers. The variant was confirmed using Sanger sequencing (Figure 3). This variant has been identified as pathogenic according to the Human Gene Mutation Database.

## Discussion

This study describes the third Japanese case of CC2L, in which MRS of normal-appearing white matter revealed reduced Cho levels for the first time. Relatively common clinical manifestations of CC2L include cerebellar ataxia, headache, and visual changes. These neurological deficits are often mild, stable, slowly progressive, and nonspecific [2]; therefore, CC2L might be undiagnosed. The characteristic MRI findings in CC2L are abnormal signals (T2 prolongation and reduced diffusion) in the posterior limbs of the internal capsules, cerebral peduncles, and middle cerebellar peduncles [1]. Our patient was asymptomatic until her 20s, when she presented with headache, vertigo, visual impairment at night, and ataxic gait, and these characteristic MRI findings led to the probable diagnosis of CC2L.

To our knowledge, all three Japanese patients with CC2L, including the present patient, carried the same variant, namely a homozygous variant (c.61dup, p.(Leu21Profs*27)) of *CLCN2*, which has never been reported outside Japan [2-4]. The first case involved a 22-month-old girl who presented with seizures at three months, but her subsequent psychomotor development was normal. MRI at three, seven, and 13 months revealed characteristic findings of CC2L [3]. The second case involved a six-year-old child with persistent headaches after aseptic meningitis, and an MRI revealed a bright tree appearance (BTA). After the headache resolved, the BTA disappeared on MRI, but the characteristic findings of CC2L remained [4]. Both patients unintentionally exhibited findings characteristic of CC2L on MRI, because they subsequently had normal development with no sequelae. Our patient also had normal development and no abnormal neurological findings in childhood. The allele frequency of this variant is 0.0007 (31 of 44,868 alleles) in East Asians, and it is not recognized in other regions based on the Genome Aggregation Database v4.0.0 (https://gnomad.broadinstitute.org/). However, its allele frequency in Japanese is 0.002 (227 of 108,604 alleles) based on the Japanese Multi-Omics Reference Panel (https://jmorp.megabank.tohoku.ac.jp/). Therefore, this variant might be relatively common in the Japanese population. According to the database, approximately one in 250 Japanese people are heterozygous carriers, and one in 250,000 Japanese people have CC2L. Many cases of CC2L could be undiagnosed because they remain asymptomatic and lack a specific clinical manifestation that could prompt evaluation by brain MRI.

CLCN2 encodes chloride channel-2 (ClC-2), which is widely expressed in various tissues, especially in the brains of humans, and plays an important role in ion homeostasis [1]. CLCN2-knockout mice display a leukodystrophy phenotype with extensive myelin vacuolization [5]. Homozygous variants in the *CLCN2* gene cause the dysfunction of its encoded ClC-2 chloride channel protein, leading to intramyelinic edema [6], which results in reduced diffusion on diffusion-weighted imaging and prolonged latency of the P100 wave, reflecting optic nerve dysfunction.

We previously reported that reduced Cho levels on MRS might represent a common marker for hypomyelination disorders [7]. Reduced Cho content on MRS has also been reported in white matter lesions of adenosine kinase deficiency, in which intramyelinic edema is presumed to be the etiology [8]. In CC2L, Cho levels are presumed to be similarly reduced in MRS because of intramyelinic edema. Only one report has described MRS findings associated with *CLCN2* variants. In the report, an 18-month-old child had normal-for-age Cho levels (as analyzed by a semi-quantitative method) [6], but no quantitative analysis was conducted. In our case, MRS revealed reduced Cho levels in white matter that appeared normal on MRI. We evaluated the Cho concentration using LCModel, which revealed reduced levels of white matter with a normal appearance on MRI. This study demonstrated decreased Cho levels in intramyelinic edema associated with CC2L for the first time and MRS appeared more sensitive in detecting intramyelinic edema than MRI signal changes.

## Conclusions

We described the third case of CC2L in Japan. A homozygous variant (c.61dup, p.(Leu21Profs*27)) of *CLCN2* has never been reported outside Japan, but it is relatively common in the Japanese population. MRS of the normal-appearing white matter revealed reduced Cho levels. The MRS findings might reflect intramyelinic edema, and MRS appears to be more sensitive than MRI signal changes in CC2L.
